# Characterization of the sesame (*Sesamum indicum *L.) global transcriptome using Illumina paired-end sequencing and development of EST-SSR markers

**DOI:** 10.1186/1471-2164-12-451

**Published:** 2011-09-19

**Authors:** Wenliang Wei, Xiaoqiong Qi, Linhai Wang, Yanxin Zhang, Wei Hua, Donghua Li, Haixia Lv, Xiurong Zhang

**Affiliations:** 1 Key Laboratory of Oil Crops Biology of the Ministry of Agriculture, Sesame Germplasm and Genetic Breeding Laboratory, Oil Crops Research Institute of Chinese Academy of Agricultural Sciences (OCRI-CAAS), Wuhan, 430062, China

## Abstract

**Background:**

Sesame is an important oil crop, but limited transcriptomic and genomic data are currently available. This information is essential to clarify the fatty acid and lignan biosynthesis molecular mechanism. In addition, a shortage of sesame molecular markers limits the efficiency and accuracy of genetic breeding. High-throughput transcriptomic sequencing is essential to generate a large transcriptome sequence dataset for gene discovery and molecular marker development.

**Results:**

Sesame transcriptomes from five tissues were sequenced using Illumina paired-end sequencing technology. The cleaned raw reads were assembled into a total of 86,222 unigenes with an average length of 629 bp. Of the unigenes, 46,584 (54.03%) had significant similarity with proteins in the NCBI nonredundant protein database and Swiss-Prot database (E-value < 10^-5^). Of these annotated unigenes, 10,805 and 27,588 unigenes were assigned to gene ontology categories and clusters of orthologous groups, respectively. In total, 22,003 (25.52%) unigenes were mapped onto 119 pathways using the Kyoto Encyclopedia of Genes and Genomes Pathway database (KEGG). Furthermore, 44,750 unigenes showed homology to 15,460 *Arabidopsis *genes based on BLASTx analysis against The Arabidopsis Information Resource (TAIR, Version 10) and revealed relatively high gene coverage. In total, 7,702 unigenes were converted into SSR markers (EST-SSR). Dinucleotide SSRs were the dominant repeat motif (67.07%, 5,166), followed by trinucleotide (24.89%, 1,917), tetranucleotide (4.31%, 332), hexanucleotide (2.62%, 202), and pentanucleotide (1.10%, 85) SSRs. AG/CT (46.29%) was the dominant repeat motif, followed by AC/GT (16.07%), AT/AT (10.53%), AAG/CTT (6.23%), and AGG/CCT (3.39%). Fifty EST-SSRs were randomly selected to validate amplification and to determine the degree of polymorphism in the genomic DNA pools. Forty primer pairs successfully amplified DNA fragments and detected significant amounts of polymorphism among 24 sesame accessions.

**Conclusions:**

This study demonstrates that Illumina paired-end sequencing is a fast and cost-effective approach to gene discovery and molecular marker development in non-model organisms. Our results provide a comprehensive sequence resource for sesame research.

## Background

Sesame (*Sesamum indicum *L.), a member of the Pedaliaceae, is a diploid (2*n *= 26) dicotyledon and one of the oldest oil seed crops, growing widely in tropical and subtropical areas [1,2]. Sesame seeds are an important source of oil (44-58%), protein (18-25%), and carbohydrates (13.5%) [3], and are traditionally consumed directly. They are used as active ingredients in antiseptics, bactericides, viricides, disinfectants, moth repellants, and antitubercular agents because they contain natural antioxidants such as sesamin and sesamolin [4]. Among the primary edible oils, sesame oil has the highest antioxidant content [5] and contains abundant fatty acids such as oleic acid (43%), linoleic acid (35%), palmitic acid (11%), and stearic acid (7%) [3]. In addition, sesame oil is important in the food industry because of its distinct flavor. These characteristics have stimulated interest in the biochemical and physiological composition of sesame oil [6].

Previous studies on sesame have mainly focused on quantitative genetics [7], traditional genetic breeding [8], and genetic relationships and diversity among sesame germplasm collections [9,10]. Although much effort has been devoted to cloning key genes and characterizing fatty acid elongation and unsaturated fatty acid biosynthesis in sesame [11-13], the molecular mechanisms behind fatty acid biosynthesis and metabolism remain unclear. Publicly available datasets are of limited use for future sesame research, such as elucidating the molecular mechanisms of specific traits and understanding the complexity of the transcriptome, gene expression regulation, and gene networks. Progress in novel gene discovery and molecular breeding in sesame has been limited by the lack of genomic information. For example, only 3,328 expressed sequence tag (EST) sequences in sesame have been deposited in the dbEST GenBank database (as at January 2011).

Molecular markers play an important role in many aspects of plant breeding, such as identification of the genes responsible for desirable traits. Molecular markers have been widely used to map important genes and assist with the breeding of oil crops. However, in sesame, only 10 genomic simple sequence repeat (SSR) [14] and 44 EST-SSR [15] markers have been developed. Genetic relationships and diversity among germplasm collections have been investigated mostly using AFLP, ISSR, and RAPD markers. In sesame, marker-assisted selection and molecular breeding lag behind other crops owing to a lack of effective molecular markers. Thus, a rapid and cost-effective approach to develop molecular markers for sesame is required. Compared with other types of molecular markers, SSRs have many advantages, such as simplicity, effectiveness, abundance, hypervariability, reproducibility, codominant inheritance, and extensive genomic coverage [16]. Based on the original sequences used to identify simple repeats, SSRs can be divided into genomic SSRs and EST-SSRs. Traditional methods to isolate and identify genomic SSRs are costly, labor-intensive, and time-consuming [17,18]. In addition, the interspecific transferability of genomic SSRs is limited because of either a disappearance of the repeat region or degeneration of the primer binding sites [19]. Alternatively, EST-SSRs are derived from expressed sequences, which are more evolutionary conserved than noncoding sequences; therefore, EST-SSR markers have a relatively high transferability. With the increasing number of ESTs deposited in public databases, an expanding number of EST-SSRs have been developed, and the polymorphism and transferability of EST-SSRs have been evaluated in many plant species [20-30].

The transcriptome is the complete set and quantity of transcripts in a cell at a specific developmental stage or under a physiological condition. The transcriptome provides information on gene expression, gene regulation, and amino acid content of proteins. Therefore, transcriptome analysis is essential to interpret the functional elements of the genome and reveal the molecular constituents of cells and tissues. Transcriptome or EST sequencing is an efficient way to generate functional genomic-level data for non-model organisms. Large collections of EST sequences are invaluable for gene annotation and discovery [31,32], comparative genomics [33], development of molecular markers [34,35], and population genomics studies of genetic variation associated with adaptive traits [36]. Recently, an increasing number of EST datasets have become available for model and non-model organisms, but relatively few ESTs are currently available for sesame.

Numerous technologies have been developed to analyze and quantify the transcriptome. Initially, a traditional sequencing method was used, but this approach is costly, time-consuming, and sensitive to cloning biases since it involves cDNA library construction, cloning, and labor-intensive Sanger sequencing. Because of the deep coverage and single base-pair resolution provided by next-generation sequencing instruments, RNA sequencing (RNA-seq) is an efficient method to analyze transcriptome data. Theoretically, any high-throughput sequencing technology can be used for RNA-seq, such as the Illumina Genome Analyzer, Applied Biosystems' SOLiD, and Roche 454 Life Sciences system. Because of the increased read length by 454 pyrosequencing compared to the other two platforms [37-39], the 454 system is usually adopted for non-model organisms to create a transcriptome database [39], and a short-read-based technology such as the Solexa platform has been used for resequencing [40]. Recent algorithmic [41] and experimental (e.g., Illumina/Solexa mate-pair and short-read paired-ends libraries) advances are likely to increase the applicability of Illumina sequencing and *de novo *assembly, which has been successfully and increasingly used for model [40,42-44] and non-model organisms [39,45-47]. These technologies are efficient, inexpensive, and reliable for genome and transcriptome sequencing, and suitable for non-model organisms such as sesame.

In this study, we sampled the pooled transcriptomes of roots, leaves, shoot tips, flowers, and the developing seeds of sesame using Illumina paired-end sequencing technology to generate a large-scale EST database and develop a set of EST-SSRs. To our knowledge, this study is the first to characterize the complete transcriptome of sesame by analyzing large-scale transcript sequences using an Illumina paired-end sequencing strategy. These EST datasets will serve as a valuable resource for novel gene discovery and marker-assisted selective breeding in sesame.

## Results

### Illumina paired-end sequencing and *de novo *assembly

To obtain a global overview of the sesame transcriptome and gene activity at nucleotide resolution, RNA was extracted from five different sesame tissues including the roots, leaves, flowers, developing seeds, and shoot tips, and equally mixed. To minimize systematic bias from transcriptome sampling and Illumina sequencing, and to enhance the accuracy of detecting low-abundance transcripts, three cDNA libraries from the same pooled RNA sample were constructed and sequenced separately using an Illumina HiSeq2000 genome analyzer.

**Figure 1:**
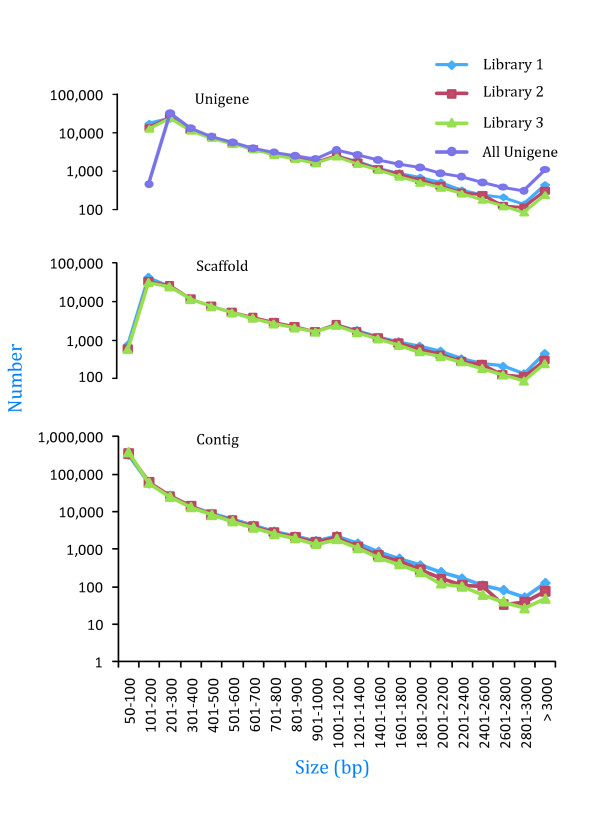
**Size distribution of the assembled contigs, scaffolds, and unigenes in the three libraries**.

Each sequenced sample yielded 2 × 90-bp independent reads from either end of a cDNA fragment. After stringent quality assessment and data filtering, 26,266,670 reads in each library with 95.34% Q20 bases (those with a base quality greater than 20) were selected as high-quality reads for further analysis. Using the SOAPdenovo assembly program [42], next-generation short-read sequences in libraries 1, 2, and 3 were assembled into 461,579, 487,989, and 500,924 contigs, respectively. The frequency distribution of these contigs in each library is shown in Figure 1. The contig length showed little difference between libraries; the average contig size was 170 bp, 152 bp, and 158 bp, and the median contig length (N50) was 220 bp, 150 bp, and 180 bp in libraries 1, 2, and 3, respectively.

Using a paired-end sequencing strategy, contigs from the same transcript can be identified and the distances between these contigs evaluated. SOAPdenovo allowed us to map the reads back to the contigs, and connect the contigs into scaffolds using 'N' to represent unknown sequences between each pair of contigs. Contigs in the three libraries were assembled into 109,263, 103,440, and 97,951 scaffolds with average lengths of 408 bp, 412 bp, and 406 bp, and with median lengths of 610 bp, 592 bp, and 576 bp in libraries 1, 2, and 3, respectively. The distribution of scaffolds is shown in Figure 1. Though 83.18%, 73.89%, and 72.84% of scaffolds did not show a gap in libraries 1, 2, and 3 (Additional file 1), respectively, 0.64 Mb, 0.91 Mb, and 0.92 Mb gaps (1.54%, 2.14%, and 2.32% of the total scaffolds in libraries 1, 2, and 3, respectively), respectively, remained unclosed.

To further shorten the remaining gaps, paired-end reads were used to fill scaffold gaps. We gathered the paired-end reads with one end mapped on the unique contig and the other located in the gap region, and filled the small gaps within the scaffolds. Sequences with the smallest number of Ns and could not be extended on either end, were defined as unigenes. At this point, more than half of the gaps were filled. For example, in library 1, only 0.20 Mb of gaps (0.49% of the total unigene sequences) remained unclosed (Additional file 1), while in libraries 2 and 3, 0.43 Mb and 0.45 Mb of gaps (1.06% and 1.21% of the total unigene sequences), respectively, remained unclosed. The *de novo *assembly in libraries 1, 2, and 3 yielded 84,546 unigenes with an average length of 490 bp, 82,709 with an average length of 484 bp, and 78,235 with an average length of 477 bp, respectively. The respective median unigene lengths in the three libraries were 671 bp, 642 bp, and 624 bp (Figure 1).

The contig, scaffold, and unigene size distributions for the three libraries were consistent (Figure 1), implying that the Illumina sequencing solution was reproducible and reliable. Therefore, unigenes from the three libraries were pooled and assembled into nonredundant unigenes for further analysis. In total, 86,222 nonredundant unigenes with a total length of 54.25 Mb, ranging from 200 bp to 12,298 bp, with an average length of 629 bp and a median length of 947 bp, were obtained. The length of 53,969 (62.59%) nonredundant unigenes ranged from 200 to 500 bp, 17,453 (20.24%) ranged from 501 to 1,000 bp, and 14,800 (17.16%) were more than 1,000 bp in length (Figure 1).

### Annotation of all nonredundant unigenes

**Figure 2:**
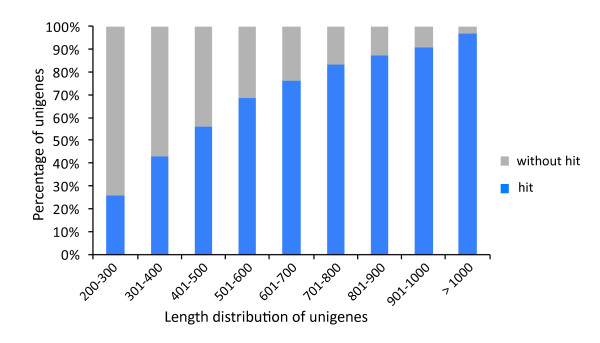
**Comparison of unigene length with or without hits**. Longer contigs were more likely to have BLASTx homologs in protein databases; 97.00% of the unigenes over 1,000 bp had BLASTx homologs, while only 25.65% of the unigenes shorter than 300 bp had homologs.

A sequence similarity search was conducted against the National Center for Biotechnology Information (NCBI), nonredundant protein (Nr) database, and Swiss-Prot protein database using the BLASTx algorithm specifying E values of less than 10^-5^. We found that 53.91% (46,479) consensus sequences showed homology with sequences in the Nr database, while 37.51% (32,345) unigenes had similarity to proteins in the Swiss-Prot database. Altogether, 54.03% (46,584) unigenes were successfully annotated in the Nr or Swiss-Prot databases. Additionally, 97.00% of the unigenes over 1,000 bp in length showed homologous matches, whereas only 25.65% of the unigenes shorter than 300 bp showed matches (Figure 2). The remaining 39,638 unigenes that had no matches in either the Nr or Swiss-Prot databases underwent gene prediction analysis using ESTScan [48], and 2,180 unigenes were identified using ORF prediction. In total, 56.56% (48,764) of the putative protein-coding unigenes were detected by homology analysis using the Nr and Swiss-Prot databases or ESTScan predictions.

To evaluate the quality and coverage of the assembled unigenes, 86,222 nonredundant unigenes were submitted to a BLASTx search against The Arabidopsis Information Resource (TAIR, Version 10), specifying an E value of less than 10^-5^. In total, 15,460 different *Arabidopsis *loci were covered by 44,750 sesame unigenes. No unigene encoding an rRNA or transposable element was detected, suggesting that rRNA or transposable element contamination did not occur in our database. The *Arabidopsis *loci were located uniformly on five chromosomes (Table 1). We further analyzed sesame unigenes and *Arabidopsis *orthologs involved in fatty acid biosynthesis and found that unigenes coded for many enzymes involved in these pathways, including fatty acid biosynthesis initiation and saturated fatty acid elongation (Table 2). These results demonstrated the reliability of Illumina paired-end sequencing and *de novo *assembly.

**Table 1:** Distribution of unigenes on *Arabidopsis *chromosomes

Chromosome	Chr 1	Chr 2	Chr 3	Chr 4	Chr 5	Chr C	Chr M	Total
**No. of hits**	3,966	2,302	3,032	2,336	3,734	55	35	15,460

**Table 2:** Unigenes involved in fatty acid biosynthesis

Query	*Arabidopsis *hit ID	Description	Score	E-value
Unigene37331	AT5G16390.1	chloroplastic acetylcoenzyme A carboxylase 1	154	6.00E-38
Unigene40848	AT5G35360.3	acetyl Co-enzyme a carboxylase biotin carboxylase subunit	877	0
Unigene3230	AT2G38040.2	acetyl Co-enzyme a carboxylase carboxyltransferase alpha subunit	202	3.00E-53
Unigene3676	ATCG00500.1	acetyl-CoA carboxylase carboxyl transferase subunit beta	451	1.00E-127
Unigene39950	AT1G36180.1	acetyl-CoA carboxylase 2	1559	0
Unigene33384	AT2G30200.1	catalytics; transferases; [acyl-carrier-protein] S-malonyltransferases; binding	543	1.00E-154
Unigene10471	AT1G62640.2	3-ketoacyl-acyl carrier protein synthase III	550	1.00E-156
Unigene6985	AT1G74960.3	fatty acid biosynthesis 1	35.4	9.00E-03
Unigene23348	AT5G46290.1	3-ketoacyl-acyl carrier protein synthase I	494	1.00E-139
Unigene7472	AT2G04540.1	beta-ketoacyl synthase	370	1.00E-103
Unigene10471	AT1G62640.2	3-ketoacyl-acyl carrier protein synthase III	550	1.00E-156
Unigene5774	AT1G24360.1	NAD(P)-binding Rossmann-fold superfamily protein	79.3	5.00E-16
Unigene637	AT2G22230.1	thioesterase superfamily protein	304	1.00E-82
Unigene15731	AT2G05990.2	NAD(P)-binding Rossmann-fold superfamily protein	545	1.00E-155
Unigene42291	AT3G25110.1	fatA acyl-ACP thioesterase	104	5.00E-23
Unigene2384	AT1G08510.1	fatty acyl-ACP thioesterases B	58.5	2.00E-09
Unigene8278	AT3G12120.2	fatty acid desaturase 2	134	2.00E-32

**Figure 3:**
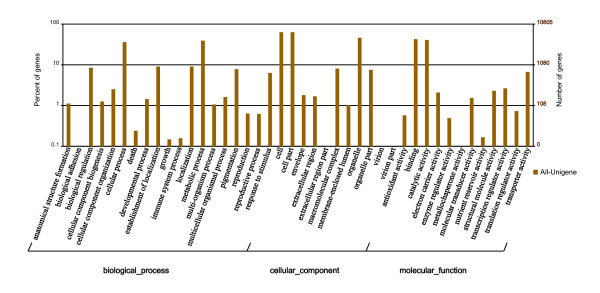
**Gene ontology classification of assembled unigenes**. The results are summarized into three main categories: biological processes, cellular components, and molecular functions. In total, 10,805 unigenes with BLASTx matches were assigned to gene ontologies.

Based on Nr annotation, 10,805 unigenes were assigned gene ontology (GO) terms. GO-annotated unigenes belonged to the biological processes, cellular components, and molecular functions clusters and were distributed across more than 40 categories, including biochemistry, metabolism, growth, development, and apoptosis (Figure 3). Among the biological processes category, metabolic processes (39.23%) was the most dominant group, followed by cellular processes (36.07%), localization (9.11%), and establishment of localization (9.06%) (Figure 3). Regarding molecular functions, 42.60% of the unigenes were assigned to binding, followed by catalytic activity (40.81%), transporter activity (6.71%), and transcription regulator activity (2.66%) (Figure 3). Among the cellular components category, cell (63.49%) and cell part (63.47%) were the dominant groups, followed by organelles (46.36%) and macromolecular complexes (8.10%) (Figure 3).

**Figure 4:**
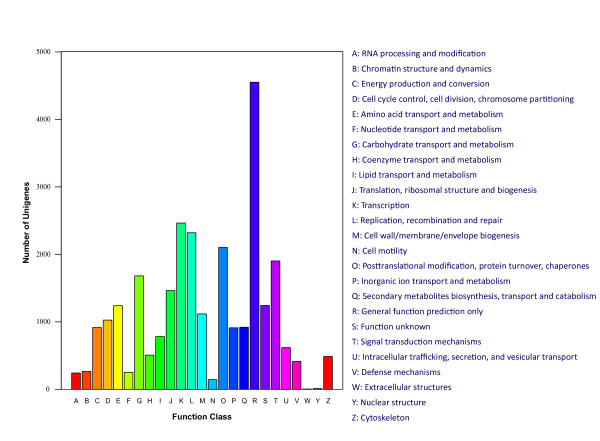
**Clusters of orthologous groups (COG) classification**. In total, 27,588 of the 46,479 sequences with Nr hits were grouped into 25 COG classifications.

In addition, all unigenes were subjected to a search against the Cluster of Orthologous Groups (COG) database for functional prediction and classification. Overall, 27,588 of the 46,479 sequences showing Nr hits were assigned to COG classifications (Figure 4). COG-annotated putative proteins were functionally classified into at least 25 molecular families such as cellular structure, biochemistry metabolism, molecular processing, and signal transduction (Figure 4). The cluster for general function prediction (4,550; 16.49%) represented the largest group, followed by transcription (2,464; 8.93%), replication, recombination and repair (2,321; 8.41%), posttranslational modification, protein turnover and chaperones (2,103; 7.62%), signal transduction mechanisms (1,903, 6.90%), carbohydrate transport and metabolism (1,681, 6.09%) and translation, ribosomal structure and biogenesis (1,464; 5.31%), whereas only a few unigenes were assigned to nuclear structure and extracellular structure (11 and 5 unigenes, respectively). In addition, 781 unigenes were assigned to lipid transport and metabolism (Figure 4).

### Reconstruction of oil accumulation metabolic pathways

According to the Kyoto Encyclopedia of Genes and Genomes (KEGG) database, 22,003 unigenes were grouped into 119 cellular metabolic or signaling pathways including cellular growth, differentiation, apoptosis, migration, endocrine, and numerous biosynthesis metabolic pathways (Additional file 2). Being an important oil crop, previous research has focused mostly on fatty acid and lipid metabolism pathways. Unigenes encode the majority of enzymes in the fatty acid biosynthesis pathway. Specifically, unigenes encoded enzymes for the biosynthesis of oleic acid, stearic acid (FatA), and palmitic acid (FatA and FatB), the main constituents of sesame seed oil. Additionally, six ESTs (unigene8278, unigene34351, unigene15844, unigene17939, unigene27262, and unigene31569) encoded oleoyl-ACP desaturase (FAD2, EC:1.14.19.-), which catalyzes polyunsaturation of oleoyl-ACP (18:1) to linoleoyl-ACP (18:2). Since oleic and linoleic acids are the major components of sesame oil, FAD2 is a potential biological target to modulate sesame oil composition.

### Frequency and distribution of EST-SSRs in the sesame transcriptome

In total, 6,276 sequences containing 7,702 SSRs were identified from 86,222 consensus sequences, with 1,104 of the EST sequences containing more than one SSR (Additional file 3). The EST-SSR frequency in the sesame transcriptome was 8.93%, and the distribution density was 141.97 per Mb. The most abundant type of repeat motif was dinucleotide (67.07%), followed by trinucleotide (24.89%), tetranucleotide (4.31%), hexanucleotide (2.62%), and pentanucleotide (1.10%) repeat units (Table 3). The frequencies of EST-SSRs with different numbers of tandem repeats were calculated and are shown in Table 3. SSRs with six tandem repeats (24.82%) were the most common, followed by five tandem repeats (15.98%), seven tandem repeats (15.55%), eight tandem repeats (12.28%), and > 10 tandem repeats (11.36%). The dominant repeat motif in EST-SSRs was AG/CT (46.29%), followed by AC/GT (16.07%), AT/AT (10.53%), AAG/CTT (6.23%), and AGG/CCT (3.39%) (Table 4). However, very few CG/CG (three; 0.04%) repeats were identified in the databases.

**Table 3:** Frequency of EST-SSRs in sesame

Motif length	Repeat numbers								Total	%
			
	4	5	6	7	8	9	10	> 10		
**Di**	-	-	1,452	1,001	854	612	377	870	5,166	67.07
**Tri**	-	1,130	443	194	90	51	4	5	1,917	24.89
**Tetra**	247	65	16	2	2	0	0	0	332	4.31
**Penta**	73	11	1	0	0	0	0	0	85	1.10
**Hexa**	176	25	0	1	0	0	0	0	202	2.62
**Total**	496	1,231	1,912	1,198	946	663	381	875		
**%**	6.44	15.98	24.82	15.55	12.28	8.61	4.95	11.36		

**Table 4:** Frequency of di- and trinucleotide EST-SSR repeat motifs in sesame

Repeat motif	Repeat numbers							Total	%
			
	5	6	7	8	9	10	> 10		
**AC/GT**	-	349	247	210	148	51	133	1,138	16.07
**AG/CT**	-	786	581	512	396	302	702	3,279	46.29
**AT/AT**	-	316	172	131	68	24	35	746	10.53
**CG/CG**	-	1	1	1			0	3	0.04
**AAC/GTT**	32	20	10		1		0	63	0.89
**AAG/CTT**	237	111	50	23	19	1	0	441	6.23
**AAT/ATT**	77	26	6	2	5		0	116	1.64
**ACC/GGT**	137	55	23	13	4		1	233	3.29
**ACG/CTG**	102	35	14	8	3		0	162	2.29
**ACT/ATG**	104	46	20	13	6	1	2	192	2.71
**AGC/CGT**	79	33	12	8	5		0	137	1.93
**AGG/CCT**	135	56	30	12	5	2	0	240	3.39
**AGT/ATC**	146	40	21	11	3		2	223	3.15
**CCG/CGG**	81	21	8				0	110	1.55
**Total**	1,130	1,895	1,195	944	663	381	875		
**%**	15.95	26.75	16.87	13.33	9.36	5.38	12.35		

### Identification of polymorphic markers

Fifty primer pairs (designated ZM_1-ZM_50) were randomly selected from the 7,702 microsatellites to evaluate their application and the polymorphism across 24 sesame accessions (Additional file 4). Forty-five of the 50 primer pairs successfully amplified fragments. Among the 45 successful primer pairs, 40 produced PCR amplicons at the expected size and five generated PCR fragments longer than expected. The majority of the 40 microsatellite loci showed allelic polymorphism. The number of alleles per locus varied from four to 18 (mean: 6.55). The observed mean heterozygosity (*Ho*) was 0.84, while the expected value (*He*) was 0.76 (Table 5). Polymorphism information content (PIC) values ranged from 0.46 to 0.82 (mean: 0.70).

**Table 5:** Characterization of 40 EST-SSRs among 24 sesame accessions

Primer	SSRs	Forward primer (5'-3')	Reverse primer (5'-3')	No. of alleles	*Ho*	*He*	PIC
ZM_1	(CA)6	GTTTCTTGGTCTTATCACAGC	TACCAACGTCACTCTTCTTTC	5	1.00	0.72	0.66
ZM_2	(AC)8	CTTCTTGAAGTTCTGGTGTTG	ATTCTTGGAGAAAGAGTGAGG	6	0.91	0.77	0.72
ZM_3	(GT)8	ATCACCACACACTGACACAG	CGTGTCTGAGAATCCAATATC	7	0.96	0.79	0.74
ZM_4	(AC)8	TCCAGAGAGGAGACAATAAGA	GAGATAGATTGCGAGTTGTGT	4	0.71	0.72	0.64
ZM_5	(CT)10	GATAAAGAACTGCCAAGGAAC	CACAGCAGTGAAGAAAAGAGT	6	0.71	0.74	0.68
ZM_6	(GT)9	GGTGTGTTCTCTCTCTCACAC	GGGCTGCTCAATAAATGTAG	6	0.75	0.78	0.73
ZM_7	(AGC)6	ATCCTCTGCTCCTAACTTCAT	TCTGGTACTATCCTCAAGCAA	6	0.96	0.79	0.74
ZM_8	(AG)6	TCTCTCTCTCTCTCGTTCTTG	CCCACTGTACCTCTCCATATT	5	0.70	0.73	0.66
ZM_9	(AAT)5	CTCGCCGTTCTAACATTATC	AGTACAGTCTCCTCCGATTTT	5	0.50	0.72	0.65
ZM_10	(CTTCCT)4	ATGCCCATCTCCATATACTCT	AATTCTTGCCTGACTCTACG	8	0.83	0.81	0.76
ZM_11	(CT)12	GGATTCTCTAGACATGGCTTT	AACGCAGAATTCTCTCCTACT	8	1.00	0.85	0.81
ZM_12	(CT)7	ATTGCTGTGCAATCCTTATC	ATCTCTTTCTACCACCACGTT	5	1.00	0.79	0.74
ZM_13	(GA)7	GCAGAAGGCAATAAAGTCAT	GCGTCAGAAGAAAAATACTGG	7	1.00	0.80	0.75
ZM_14	(ATC)5	GGAAGGCGAGTTGATAGATAA	CATGGGATGTTCAAAGAACT	6	0.96	0.79	0.73
ZM_15	(GA)7	ACTCTATCACCGAGTGGAGAC	CTACCCTTTTCCTCGTAGC	7	0.65	0.75	0.69
ZM_16	(GATC)4	AGGTAGAATTACATGCTGTGC	GCTTCCTCCTTCATTCATATC	5	0.60	0.71	0.65
ZM_17	(CT)6	CTTGCTTCCTCTTTTCTCTCT	ACACTGTACTCAGCGGATTT	5	1.00	0.79	0.72
ZM_18	(CT)6	AATACCCTTCAGTATTCAGGTG	CAACAACACAAACACTGCTAC	5	1.00	0.80	0.75
ZM_20	(GAG)5	GGGATGTTGATAGAGATGTTG	TCTTTCACTCTCACACACACA	10	1.00	0.86	0.82
ZM_21	(AC)7	CTCTCTCTCTCTGCTGTTTCA	GCCATACGATCTCAAAATCAC	8	0.95	0.86	0.82
ZM_22	(AT)9(GA)6	ACCACCGATCTACTCACTTTT	CCACTGCACACTACAGTTTTT	9	0.68	0.86	0.82
ZM_23	(AT)7(GA)8	CGTATGTCAAGATGAAGCAGT	ATCAACAATTCCACTCAACC	6	0.95	0.75	0.69
ZM_24	(TC)6(CCATTT)4	CCACACTCAAAACCAAGAAA	GCGAAGAGATTATATACACACG	5	0.75	0.72	0.66
ZM_25	(AT)6	CCTGAACCTTCTCTCTCTCTC	ACTGACAGTACGAATTCACCA	4	0.58	0.71	0.64
ZM_26	(GA)8	ACTTCAACTTCAACCTCAACC	TGTGCATAAAAACCCTCTCT	5	1.00	0.65	0.57
ZM_29	(AG)9	CATTACAATAGCCCGAAAAG	TACTGTTCCTCCTCCTCTCTT	6	1.00	0.70	0.62
ZM_30	(TAT)5	CACTCCACTCATTATCCAAAG	CAAGACACAACTGACACGTAA	6	1.00	0.77	0.72
ZM_31	(GA)7	GAGCACTCTCTTCTCTCTTCC	AAAAGAGGATGGCAACTGTA	5	0.78	0.72	0.65
ZM_32	(GCC)5	CACGAAGAGTGAGAGAGAGAG	CTACCAAAAGTCCCTGAATCT	5	0.77	0.70	0.62
ZM_33	(TCA)5	GAGACAGTACACTTGGGACAA	CTCTTCTTGGGCATTAACTCT	6	0.74	0.73	0.67
ZM_34	(AG)14	AAGTCCCTTTTCAAGCAATC	GAGAGAGGAAAATGCAGAGAG	10	0.79	0.83	0.79
ZM_35	(TTCC)4	AATGCATAGTGCATAGGGTAG	TGGAAAGTAGAGATCGCATAG	6	0.96	0.75	0.69
ZM_38	(GA)8	CAGCTTCCTGATTTGATTTG	AGATTGCAAGAATCGCTTAG	5	0.85	0.69	0.62
ZM_39	(TCA)5	AGAGGCAGAGGAGTTGATAAT	CTTAACTGTAACTCCCTTTTCG	6	0.81	0.78	0.72
ZM_40	(ACTCCA)5	CGAAAAGGGAGTTACAGTTAAG	CTTCCTCTCCTATCATCCTGT	7	0.82	0.82	0.77
ZM_41	(AC)8	GATATGATTCAAACCCCTCAG	CTTCTGCACTACCATCAATTC	5	0.63	0.55	0.46
ZM_43	(ACAT)4	CTTGGATATCAGTTTCCTGTG	GTTCTCCACAGTCAAAACACT	7	0.89	0.72	0.65
ZM_44	(TC)9	GTCTTAAGCCCTCTTAGTTCC	GAAAACCTTCAATGTCAGGA	7	0.77	0.77	0.73
ZM_45	(TA)6	GCAAAATCTCTGTTGTCTCAG	GTGTTCCTACCACTCAACACA	18	0.83	0.83	0.80
ZM_47	(TC)8	GTTTCCAGGTCTATTCCTTTG	AGGTAGAGCTAATCCTTACCG	10	0.71	0.83	0.79
Mean				6.55	0.84	0.76	0.70

## Discussion

### Illumina paired-end sequencing

Transcriptome sequencing is an important tool for expression pattern identification and gene discovery. Numerous technologies have been developed to analyze and quantify the transcriptome. For example, traditional EST sequencing methods, such as Sanger sequencing, have made significant contributions to current genomics research and dbEST database construction, but this approach is costly, time-consuming, and sensitive to cloning biases. Next-generation sequencing (NGS) technologies present opportunities for plant genomic analyses with or without a complete genome sequence. Because of the potential for high throughput, accuracy, and the low cost, NGS is widely applied to analyze transcriptomes qualitatively and quantitatively, and has been used successfully for *de novo *transcriptome sequencing and assembly in many organisms [33,39,42-47,49].

Only 3,328 ESTs from a cDNA library for developing sesame seeds (5-25 days after pollination) have been deposited in NCBI dbEST databases [50]. In the present study, a transcriptome sequencing analysis of mixed RNA from five sesame tissues (root, leaf, flower, developing seed, and shoot tip) was conducted using the Illumina platform. Six Gbp of data were generated and assembled into unigenes. This large number of reads with paired-end information produced much longer unigenes (mean: 629 bp) than those in previous studies [35,37,49,51-53]. This increased transcriptome nucleotide coverage depth facilitated *de novo *assembly, enhanced the sequencing accuracy, and avoided possible contamination. For example, no transposable element contamination was detected in our database. The unigenes were subjected to BLASTx analysis against the TAIR Version 10 database, and 44,750 unigenes showed homology to 15,460 *Arabidopsis *genes. Moreover, our database revealed that unigenes encoded for the majority of enzymes involved in fatty acid biosynthesis (Table 2), suggesting that relatively short reads from Illumina paired-end sequencing for a non-model organism can be effectively and accurately assembled.

In our study, 54.03% (46,584 of 86,222) of the sesame unigenes had homologs in the Nr or Swiss-Prot protein databases, whereas in *Epimedium sagittatum *[49], whitefly [46], and sweet potato [52], only 38.50%, 16.20%, and 46.21% unigenes, respectively, had homologs in the Nr database. The average unigene length in our database was 629 bp, while the length in the above three databases was 246 bp, 266 bp, and 581 bp, respectively. The higher percentage of hits found in our study was partially a result of the increased number of long sequences in our unigene database; the results for whitefly [46] support this conclusion. Homologs in other species were not found for the remaining 45.97% (39,638) of the unique sequences. Specifically, 74.35% of unigenes shorter than 300 bp, and 3.00% of unigenes longer than 1,000 bp, showed no BLAST matches (Figure 2), which suggests that longer contigs were more likely to show BLAST hits in the protein databases. The shorter sequences may lack a characterized protein domain, or they may contain a known protein domain but not show sequence matches due to the short query sequence, resulting in false-negative results. Additionally, only limited genomic and transcriptomic information is currently available for sesame, and consequently, many sesame lineage-specific genes might not be included in current databases.

Many of the sesame unigenes were assigned to GO categories and COG classifications (Figures 3 and 4). Most representative unigenes were mapped to specific pathways, such as metabolism pathways, biosynthesis of secondary metabolites, plant-pathogen interactions, the spliceosome, and starch and sucrose metabolism, using the KEGG database (Additional file 2). Importantly, most of the genes involved in the biosynthesis of fatty acids were identified. Unigenes without BLASTx hits may function as sesame-specific genes.

Our results indicate that high-throughput RNA-seq is an efficient, inexpensive, and reliable platform for transcriptomic analysis in non-model organisms. The large number of sequences generated in this study provides valuable sequence information at the transcriptomic level for novel gene discovery, or for the investigation of sesame molecular mechanisms.

### EST-SSR frequency and distribution in the sesame transcriptome

Previously, genetic diversity analysis of sesame germplasm has mostly depended on AFLP, ISSR, and RAPD markers [9,10,54-56]. Polymorphic SSR markers play an important role in genetic diversity research, population genetics, linkage mapping, comparative genomics, and association analysis. In the present study, 7,702 perfect microsatellites exceeding 12 bp were identified from the sesame EST dataset, and 8.93% of the EST sequences possessed SSRs. The SSR frequency in this study is consistent with the range of frequencies reported for other dicotyledonous species (2.65-16.82%) [57]. The EST-SSR frequency is dependent on several factors such as genome structure or composition [58], arithmetical method for SSR detection, and the parameters for exploration of microsatellites.

Dinucleotide repeats were the most frequent SSR motif type. This finding is consistent with results reported for *Arabidopsis*, peanut, canola, sugar beet, cabbage, soybean, sunflower, sweet potato, pea, and grape [57], whereas trinucleotide repeats were the most abundant class of SSRs in cereals such as rice, wheat, and barley [59]. Among the dinucleotide repeats, AG/CT (46.29%) was the most frequent motif in our dataset, whereas CG/CG (0.04%) motifs were very rare. Among the trinucleotide repeats, the AAG/CTT motif was common (6.23%) among the microsatellites. Our results are consistent with those for other plant species [49,57,58,60,61]. In plants, TC and CTT repeats are typically found in transcribed regions and occur at a high frequency in 5' UTRs; CT microsatellites in 5' UTRs may be involved in antisense transcription and play a role in gene regulation [62].

### EST-SSR marker polymorphism

The majority of sesame EST-SSRs generated high-quality amplicons, suggesting that ESTs are suitable for specific primer design. In this study, 45 (90%) of the primer pairs designed from ESTs successfully yielded amplicons. Among the successful primer pairs, 40 of the 45 amplicons were of the expected size. The deviation of five amplicons from the expected size may have been due to the presence of introns [63,64], large insertions or repeat number variations, a lack of specificity, or assembly errors. The failure of five primer pairs to produce amplicons may have been caused by the location of the primer(s) across splice sites, large introns, chimeric primer(s), or poor-quality sequences [64]. These results suggest that the assembled unigenes were of high quality and that the EST-SSRs identified in our dataset could be used in the future.

Using the EST-SSRs in our dataset, the mean number of alleles per locus (6.55) and the mean *He *(0.76) and *Ho *(0.84) were investigated across 24 sesame accessions. The PIC values ranged from 0.46 to 0.82 (mean: 0.70). The difference between *He *and *Ho *at all loci may be the result of a very high self-pollination rate within the population. These findings indicated that polymorphism was relatively high, which is corroborated by sesame genomic SSRs [14]. Since we identified 7,702 SSRs in our dataset, more PCR primers could be designed in the future as tools for germplasm polymorphism assessment, quantitative trait loci mapping, and functional gene cloning in sesame.

## Conclusions

In this study, a large EST dataset composed of 86,222 unigenes derived from the sesame transcriptome was assembled. These results indicated that Illumina paired-end sequencing is a fast and cost-effective approach to novel gene discovery and molecular marker development in non-model organisms. Based on the generated sequences, 7,702 EST-SSRs were identified and characterized as potential molecular markers. Fifty primer pairs were randomly selected to detect polymorphism among 24 sesame accessions, and 40 (80%) of these primer pairs successfully amplified fragments, revealing abundant polymorphism. The EST-SSR markers developed in this study can be used for construction of high-resolution genetic linkage maps and to perform gene-based association analyses in sesame. To our knowledge, this is the first application of Illumina paired-end sequencing technology to investigate the whole transcriptome of sesame and to assemble RNA-seq reads without a reference genome. The dataset will improve our understanding of the molecular mechanisms of fatty acid biosynthesis, lignan biosynthesis, and other biochemical processes in sesame.

## Methods

### Sample collection and preparation

Sesame cv. Zhongzhi No. 11 was grown at the experimental station of the Oil Crops Research Institute, Chinese Academy of Agricultural Sciences, Wuhan, China. Young roots, leaves, flowers, developing seeds, and shoot tips of plants at anthesis were collected, frozen immediately in liquid nitrogen, and stored at -70°C until use.

### RNA extraction and library preparation for transcriptome analysis

Total RNA was isolated using the TRIzol reagent according to the manufacturer's instructions (Invitrogen). The total RNA concentration was quantified using an ultraviolet (UV) spectrophotometer, and RNA quality was assessed on 1.0% denaturing agarose gels. Equal volumes of RNA from each of the five tissues were pooled. The mixed RNA extract was subjected to Solexa sequencing analysis at the Beijing Genomics Institute (BGI; Shenzhen, China). RNA quality and quantity were verified using a NanoDrop 1000 spectrophotometer and an Agilent 2100 Bioanalyzer prior to further processing at BGI. The total RNA was treated with DNase I prior to library construction, and poly-(A) mRNA was purified with Magnetic Oligo (dT) Beads. The mRNA was fragmented by treatment with divalent cations and heat. The cleaved RNA fragments were transcribed into first-strand cDNA using reverse transcriptase and random hexamer-primers, followed by second-strand cDNA synthesis using DNA polymerase I and RNaseH. The double-stranded cDNA was further subjected to end-repair using T4 DNA polymerase, the Klenow fragment, and T4 polynucleotide kinase followed by a single < A > base addition using Klenow 3' to 5' exo-polymerase, then ligated with an adapter or index adapter using T4 DNA ligase. Adaptor-ligated fragments were separated by size on an agarose gel, and the desired range of cDNA fragments (200 ± 25 bp) were excised from the gel. PCR was performed to selectively enrich and amplify the cDNA fragments. After validation with an Agilent 2100 Bioanalyzer and ABI StepOnePlus Real-Time PCR System, the cDNA library was sequenced on a flow cell using an Illumina HiSeq2000 sequencing platform. In total, three duplicate cDNA libraries were constructed and sequenced separately using an Illumina HiSeq2000 genome analyzer to minimize the likelihood of systematic biases and random error in sequencing and allow for the detection of low-abundance transcripts. The sequence data were deposited in the NCBI Sequence Read Archive http://www.ncbi.nlm.nih.gov/Traces/sra under accession number SRP006700.

### Data filtering and *de novo *assembly

The cDNA library was sequenced on an Illumina HiSeq2000 sequencing platform. Image deconvolution and quality value calculations were performed using Illumina HCS 1.1 software. The raw reads were cleaned by removing adapter sequences, low-quality sequences (reads with ambiguous bases 'N'), and reads with more than 10% Q < 20 bases. *De novo *assembly of the clean reads was performed using SOAPdenovo http://soap.genomics.org.cn/soapdenovo.html with the default settings, except for the K-mer value, which was set at a specific value [42]. The best assembly was achieved with K = 29, which was chosen for de Bruijn graph construction. Although a higher K-mer value reduced the number of assembled contigs, it increased the reliability and produced longer contigs. Contigs without ambiguous bases were obtained by conjugating the K-mers in an unambiguous path. Next, the reads were mapped back to the contigs using SOAPdenovo to construct scaffolds with the paired-end information. The program detected contigs from the same transcript as well as the distances between these contigs. Next, SOAPdenovo connected the contigs between each pair of contigs using 'N' to represent unknown bases, thus generating scaffolds. Paired-end reads were used again for scaffold gap filling to obtain sequences with the least Ns and those that could not be extended at either end. Such sequences were defined as unigenes. Finally, the overlapping unigenes from three libraries were assembled into a continuous sequence using the overlapping ends of different sequences, and redundant sequences were removed to yield the maximum length nonredundant unigenes using the TIGR Gene Indices Clustering (TGICL) tools. The parameters were set at a similarity of 94% and an overlap length of 100 bp. The assembled unique sequences were deposited in the NCBI Transcriptome Shotgun Assembly database http://www.ncbi.nlm.nih.gov/genbank/TSA.html under accession numbers JL321729-JL346699, JL349641-JL382688, and JL473672-JL478462.

### Gene annotation

Unigenes were aligned with the NCBI Nr and Swiss-Prot protein databases using BLASTx with an E-value of less than 10^-5^. Unigenes that did not have homologs in these databases were scanned using ESTScan [48]. Blast2GO [65] was used to obtain GO annotation of the unigenes based on BLASTx hits against the NCBI Nr database with an E-value threshold of less than 10^-5^. WEGO [66] was used for GO functional classification of all unigenes and to plot the distribution of the sesame gene functions. The unigene sequences were also aligned to the COG database to predict and classify functions. Pathway assignments were carried out based on the KEGG database [67]. Additionally, a BLASTx search against the TAIR Version 10 database http://www.arabidopsis.org/ was performed with an E-value threshold of less than 10^-5^.

### EST-SSR detection and primer design

Potential SSR markers were detected among the 86,222 unigenes using the MISA tool http://pgrc.ipk-gatersleben.de/misa. The parameters were adjusted for identification of perfect di-, tri-, tetra-, penta-, and hexanucleotide motifs with a minimum of 6, 5, 4, 4, and 4 repeats, respectively. Mononucleotide repeats were ignored since distinguishing genuine mononucleotide repeats from polyadenylation products and single nucleotide stretch errors generated by sequencing was difficult. Primer pairs were designed using BatchPrimer3 [68]. The major parameters for primer pair design were set as follows: primer length of 18-23 bases (optimal 20 bases), PCR product size of 100-400 bp (optimal 200 bp), GC content of 40-70% (optimal 50%), and annealing temperatures of 50-60°C (optimal 55°C). Based on these parameters, 50 primer pairs were designed and synthesized for germplasm polymorphism detection in sesame.

### Survey of EST-SSR polymorphism

Twenty-four sesame accessions including Chinese landraces, cultivars, and foreign collections (Additional file 4) were selected for polymorphism investigation with the EST-SSRs. Total DNA was isolated from sesame seedlings using the CTAB method [69]. PCR amplifications were conducted in a final volume of 10 μL containing 50 ng template DNA, 1× PCR buffer, 2.0 mM MgCl_2_, 2.5 mM dNTPs, 4 μM of each primer, and 0.8 U Taq polymerase (Fermentas). The PCR reaction cycling profile was 94°C for 4 min followed by 35 cycles at 94°C for 40 s, 55°C for 40 s, 72°C for 1 min, and a final extension at 72°C for 10 min. The separation of alleles was performed on a 6% polyacrylamide gel with a 50-bp DNA marker (Promega) to calculate the length of the EST-SSR amplicons. PCR products were mixed with a half volume of loading buffer. The mixture was denatured at 95°C for 4 min before being loaded on the gel. Gels were stained with silver nitrate as previously described [70]. Perfect amplified loci were tested for polymorphism by genotyping 24 sesame accessions. The genetic diversity and mean allele number were calculated using Popgene version 1.32 [71]. Polymorphic information content (PIC) was obtained with PIC_CALC and GenAlex6 [72].

## Authors' contributions

WLW contributed to the experimental design and management, data analysis, and manuscript preparation. XQQ contributed to tissue collection, DNA extraction and EST-SSR validation, data analysis, manuscript organization and revision. LHW and WH assisted with the experimental design and reviewed the manuscript. YXZ prepared plant materials for EST-SSR validation and reviewed the manuscript, and HXL assisted with SSR experiments. DHL participated in tissue collection and RNA isolation. XRZ designed and managed the experiments, organized and reviewed the manuscript. All authors have read and approved the final manuscript.

## Supplementary Material

Additional file 1 **Gap distribution of assembled scaffolds and unigenes in three libraries**. Gap distribution (N/size) %: gap percentage (N amount/sequence) distribution. Click here for file

Additional file 2 **KEGG categories of nonredundant consensus sequences in sesame**. Click here for file

Additional file 3 **Identified EST-SSRs in sesame**. Click here for file

Additional file 4 **Sesame germplasms for polymorphism validation with EST-SSRs**. Click here for file
