# Supplementary figures and images for: Characterization of the sesame (Sesamum indicum L.) global transcriptome using Illumina paired-end sequencing and development of EST-SSR markers
Source: BMC Genomics. 2011 Sep 19;12:451. doi: 10.1186/1471-2164-12-451 (PMC3184296; doi:10.1186/1471-2164-12-451)

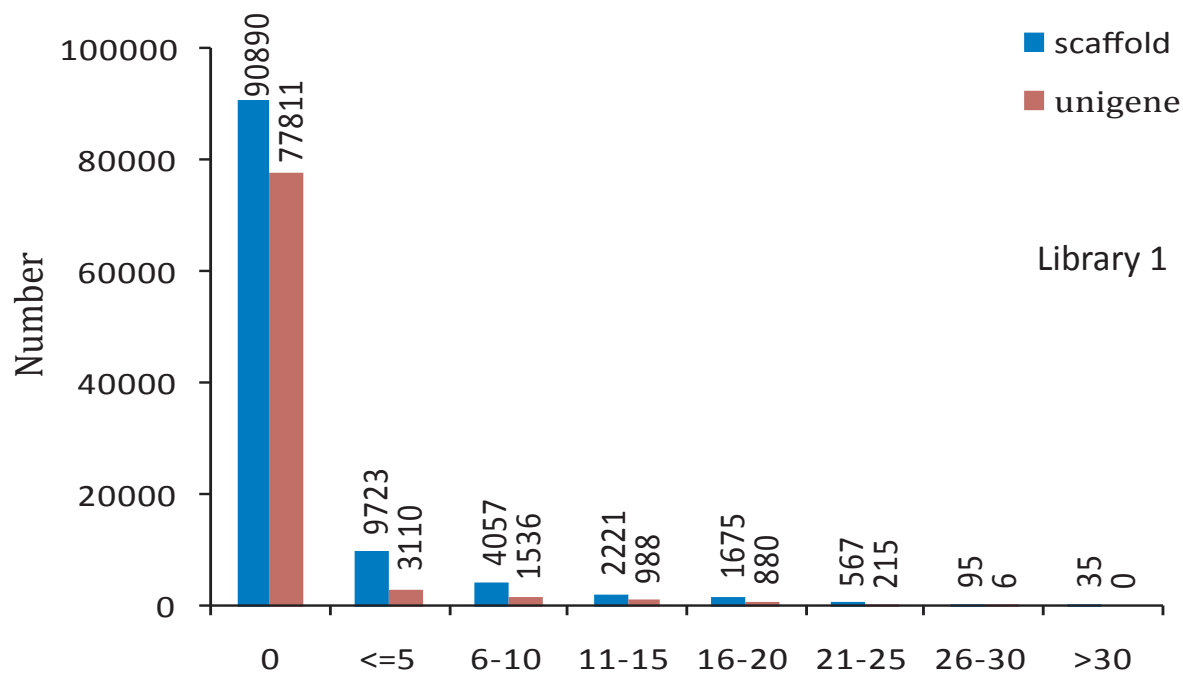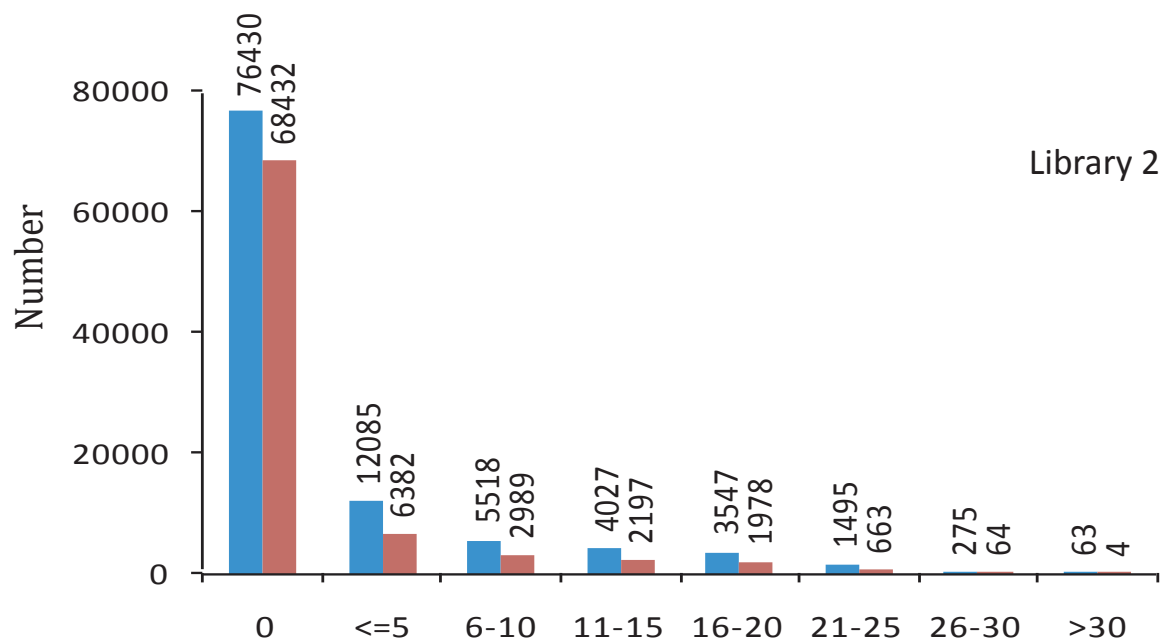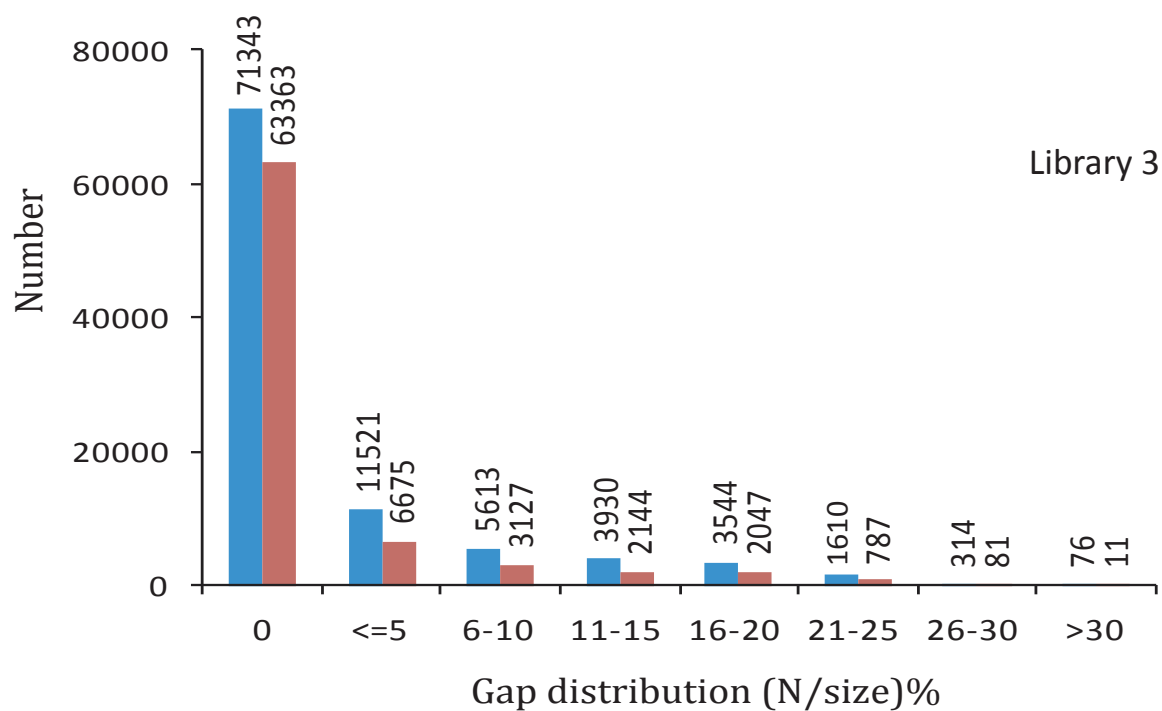

Supplement: Additional file 1 — Gap distribution of assembled scaffolds and unigenes in three libraries. Gap distribution (N/size) %: gap percentage (N amount/sequence) distribution. [file 1471-2164-12-451-S1.PDF]
