# Supplementary material for: Characterization of the sesame (Sesamum indicum L.) global transcriptome using Illumina paired-end sequencing and development of EST-SSR markers
Source: BMC Genomics. 2011 Sep 19;12:451. doi: 10.1186/1471-2164-12-451 (PMC3184296; doi:10.1186/1471-2164-12-451)
Supplement: Additional file 2 — KEGG categories of nonredundant consensus sequences in sesame. [file 1471-2164-12-451-S2.DOC]

**Additional file 2:**

**KEGG categories of nonredundant consensus sequences in sesame**

| # | Pathway | Count (22003) | Pathway ID |
| --- | --- | --- | --- |
| 1 | Metabolic pathways | 5179 | ko00950 |
| 2 | Biosynthesis of secondary metabolites | 2869 | ko03450 |
| 3 | Plant-pathogen interaction | 1777 | ko00960 |
| 4 | Spliceosome | 1169 | ko00670 |
| 5 | Starch and sucrose metabolism | 682 | ko00563 |
| 6 | Phenylpropanoid biosynthesis | 655 | ko00942 |
| 7 | Purine metabolism | 541 | ko00760 |
| 8 | Protein processing in endoplasmic reticulum | 526 | ko00770 |
| 9 | Pyrimidine metabolism | 476 | ko00590 |
| 10 | Ribosome | 473 | ko00901 |
| 11 | Ubiquitin mediated proteolysis | 456 | ko00196 |
| 12 | Oxidative phosphorylation | 432 | ko00523 |
| 13 | Stilbenoid, diarylheptanoid and gingerol biosynthesis | 392 | ko00740 |
| 14 | Endocytosis | 384 | ko00604 |
| 15 | Limonene and pinene degradation | 355 | ko00730 |
| 16 | Flavonoid biosynthesis | 351 | ko00905 |
| 17 | Cysteine and methionine metabolism | 329 | ko00902 |
| 18 | Glycolysis / Gluconeogenesis | 327 | ko00300 |
| 19 | Peroxisome | 299 | ko00790 |
| 20 | Circadian rhythm - plant | 295 | ko00430 |
| 21 | RNA degradation | 289 | ko00750 |
| 22 | Cyanoamino acid metabolism | 273 | ko00660 |
| 23 | Glycerophospholipid metabolism | 260 | ko00072 |
| 24 | Pyruvate metabolism | 256 | ko00603 |
| 25 | Amino sugar and nucleotide sugar metabolism | 255 | ko00232 |
| 26 | Phagosome | 250 | ko00785 |
| 27 | RNA polymerase | 244 | ko00780 |
| 28 | ABC transporters | 240 | ko00965 |
| 29 | Nucleotide excision repair | 239 | ko00062 |
| 30 | [Phenylalanine metabolism](../../../../I:%5CSesamum_indicum%5CSesamum_indicum%5Cannotation%5CKEGG%5CAll-Unigene.fa.htm#gene30%23gene30) | 239 | ko00950 |
| 31 | [Carbon fixation in photosynthetic organisms](../../../../I:%5CSesamum_indicum%5CSesamum_indicum%5Cannotation%5CKEGG%5CAll-Unigene.fa.htm#gene31%23gene31) | 236 | ko03450 |
| 32 | [Tryptophan metabolism](../../../../I:%5CSesamum_indicum%5CSesamum_indicum%5Cannotation%5CKEGG%5CAll-Unigene.fa.htm#gene32%23gene32) | 232 | ko00960 |
| 33 | [Zeatin biosynthesis](../../../../I:%5CSesamum_indicum%5CSesamum_indicum%5Cannotation%5CKEGG%5CAll-Unigene.fa.htm#gene33%23gene33) | 218 | ko00670 |
| 34 | [Pentose and glucuronate interconversions](../../../../I:%5CSesamum_indicum%5CSesamum_indicum%5Cannotation%5CKEGG%5CAll-Unigene.fa.htm#gene34%23gene34) | 209 | ko00563 |
| 35 | [Fatty acid metabolism](../../../../I:%5CSesamum_indicum%5CSesamum_indicum%5Cannotation%5CKEGG%5CAll-Unigene.fa.htm#gene35%23gene35) | 206 | ko00942 |
| 36 | [Galactose metabolism](../../../../I:%5CSesamum_indicum%5CSesamum_indicum%5Cannotation%5CKEGG%5CAll-Unigene.fa.htm#gene36%23gene36) | 205 | ko00760 |
| 37 | [alpha-Linolenic acid metabolism](../../../../I:%5CSesamum_indicum%5CSesamum_indicum%5Cannotation%5CKEGG%5CAll-Unigene.fa.htm#gene37%23gene37) | 196 | ko00770 |
| 38 | [Nitrogen metabolism](../../../../I:%5CSesamum_indicum%5CSesamum_indicum%5Cannotation%5CKEGG%5CAll-Unigene.fa.htm#gene38%23gene38) | 191 | ko00590 |
| 39 | [Glycerolipid metabolism](../../../../I:%5CSesamum_indicum%5CSesamum_indicum%5Cannotation%5CKEGG%5CAll-Unigene.fa.htm#gene39%23gene39) | 189 | ko00901 |
| 40 | [Phosphatidylinositol signaling system](../../../../I:%5CSesamum_indicum%5CSesamum_indicum%5Cannotation%5CKEGG%5CAll-Unigene.fa.htm#gene40%23gene40) | 187 | ko00196 |
| 41 | [Biosynthesis of unsaturated fatty acids](../../../../I:%5CSesamum_indicum%5CSesamum_indicum%5Cannotation%5CKEGG%5CAll-Unigene.fa.htm#gene41%23gene41) | 183 | ko00523 |
| 42 | [Arginine and proline metabolism](../../../../I:%5CSesamum_indicum%5CSesamum_indicum%5Cannotation%5CKEGG%5CAll-Unigene.fa.htm#gene42%23gene42) | 181 | ko00740 |
| 43 | [Fructose and mannose metabolism](../../../../I:%5CSesamum_indicum%5CSesamum_indicum%5Cannotation%5CKEGG%5CAll-Unigene.fa.htm#gene43%23gene43) | 174 | ko00604 |
| 44 | [Inositol phosphate metabolism](../../../../I:%5CSesamum_indicum%5CSesamum_indicum%5Cannotation%5CKEGG%5CAll-Unigene.fa.htm#gene44%23gene44) | 172 | ko00730 |
| 45 | [Ascorbate and aldarate metabolism](../../../../I:%5CSesamum_indicum%5CSesamum_indicum%5Cannotation%5CKEGG%5CAll-Unigene.fa.htm#gene45%23gene45) | 172 | ko00905 |
| 46 | [Alanine, aspartate and glutamate metabolism](../../../../I:%5CSesamum_indicum%5CSesamum_indicum%5Cannotation%5CKEGG%5CAll-Unigene.fa.htm#gene46%23gene46) | 170 | ko00902 |
| 47 | [DNA replication](../../../../I:%5CSesamum_indicum%5CSesamum_indicum%5Cannotation%5CKEGG%5CAll-Unigene.fa.htm#gene47%23gene47) | 169 | ko00300 |
| 48 | [Citrate cycle (TCA cycle)](../../../../I:%5CSesamum_indicum%5CSesamum_indicum%5Cannotation%5CKEGG%5CAll-Unigene.fa.htm#gene48%23gene48) | 165 | ko00790 |
| 49 | [Glycine, serine and threonine metabolism](../../../../I:%5CSesamum_indicum%5CSesamum_indicum%5Cannotation%5CKEGG%5CAll-Unigene.fa.htm#gene49%23gene49) | 162 | ko00430 |
| 50 | [Base excision repair](../../../../I:%5CSesamum_indicum%5CSesamum_indicum%5Cannotation%5CKEGG%5CAll-Unigene.fa.htm#gene50%23gene50) | 158 | ko00750 |
| 51 | [Carotenoid biosynthesis](../../../../I:%5CSesamum_indicum%5CSesamum_indicum%5Cannotation%5CKEGG%5CAll-Unigene.fa.htm#gene51%23gene51) | 157 | ko00660 |
| 52 | [Aminoacyl-tRNA biosynthesis](../../../../I:%5CSesamum_indicum%5CSesamum_indicum%5Cannotation%5CKEGG%5CAll-Unigene.fa.htm#gene52%23gene52) | 157 | ko00072 |
| 53 | [Basal transcription factors](../../../../I:%5CSesamum_indicum%5CSesamum_indicum%5Cannotation%5CKEGG%5CAll-Unigene.fa.htm#gene53%23gene53) | 153 | ko00603 |
| 54 | [Glutathione metabolism](../../../../I:%5CSesamum_indicum%5CSesamum_indicum%5Cannotation%5CKEGG%5CAll-Unigene.fa.htm#gene54%23gene54) | 153 | ko00232 |
| 55 | [Homologous recombination](../../../../I:%5CSesamum_indicum%5CSesamum_indicum%5Cannotation%5CKEGG%5CAll-Unigene.fa.htm#gene55%23gene55) | 149 | ko00785 |
| 56 | [Proteasome](../../../../I:%5CSesamum_indicum%5CSesamum_indicum%5Cannotation%5CKEGG%5CAll-Unigene.fa.htm#gene56%23gene56) | 143 | ko00780 |
| 57 | [Pentose phosphate pathway](../../../../I:%5CSesamum_indicum%5CSesamum_indicum%5Cannotation%5CKEGG%5CAll-Unigene.fa.htm#gene57%23gene57) | 142 | ko00965 |
| 58 | [Fatty acid biosynthesis](../../../../I:%5CSesamum_indicum%5CSesamum_indicum%5Cannotation%5CKEGG%5CAll-Unigene.fa.htm#gene58%23gene58) | 138 | ko00062 |
| 59 | [Terpenoid backbone biosynthesis](../../../../I:%5CSesamum_indicum%5CSesamum_indicum%5Cannotation%5CKEGG%5CAll-Unigene.fa.htm#gene59%23gene59) | 138 | ko00950 |
| 60 | [Mismatch repair](../../../../I:%5CSesamum_indicum%5CSesamum_indicum%5Cannotation%5CKEGG%5CAll-Unigene.fa.htm#gene60%23gene60) | 133 | ko03450 |
| 61 | [Flavone and flavonol biosynthesis](../../../../I:%5CSesamum_indicum%5CSesamum_indicum%5Cannotation%5CKEGG%5CAll-Unigene.fa.htm#gene61%23gene61) | 133 | ko00960 |
| 62 | [Propanoate metabolism](../../../../I:%5CSesamum_indicum%5CSesamum_indicum%5Cannotation%5CKEGG%5CAll-Unigene.fa.htm#gene62%23gene62) | 133 | ko00670 |
| 63 | [Valine, leucine and isoleucine degradation](../../../../I:%5CSesamum_indicum%5CSesamum_indicum%5Cannotation%5CKEGG%5CAll-Unigene.fa.htm#gene63%23gene63) | 132 | ko00563 |
| 64 | [Tyrosine metabolism](../../../../I:%5CSesamum_indicum%5CSesamum_indicum%5Cannotation%5CKEGG%5CAll-Unigene.fa.htm#gene64%23gene64) | 130 | ko00942 |
| 65 | [Butanoate metabolism](../../../../I:%5CSesamum_indicum%5CSesamum_indicum%5Cannotation%5CKEGG%5CAll-Unigene.fa.htm#gene65%23gene65) | 127 | ko00760 |
| 66 | [Photosynthesis](../../../../I:%5CSesamum_indicum%5CSesamum_indicum%5Cannotation%5CKEGG%5CAll-Unigene.fa.htm#gene66%23gene66) | 125 | ko00770 |
| 67 | [Linoleic acid metabolism](../../../../I:%5CSesamum_indicum%5CSesamum_indicum%5Cannotation%5CKEGG%5CAll-Unigene.fa.htm#gene67%23gene67) | 122 | ko00590 |
| 68 | [Lysine degradation](../../../../I:%5CSesamum_indicum%5CSesamum_indicum%5Cannotation%5CKEGG%5CAll-Unigene.fa.htm#gene68%23gene68) | 122 | ko00901 |
| 69 | [Porphyrin and chlorophyll metabolism](../../../../I:%5CSesamum_indicum%5CSesamum_indicum%5Cannotation%5CKEGG%5CAll-Unigene.fa.htm#gene69%23gene69) | 121 | ko00196 |
| 70 | [Natural killer cell mediated cytotoxicity](../../../../I:%5CSesamum_indicum%5CSesamum_indicum%5Cannotation%5CKEGG%5CAll-Unigene.fa.htm#gene70%23gene70) | 116 | ko00523 |
| 71 | [Sphingolipid metabolism](../../../../I:%5CSesamum_indicum%5CSesamum_indicum%5Cannotation%5CKEGG%5CAll-Unigene.fa.htm#gene71%23gene71) | 114 | ko00740 |
| 72 | [SNARE interactions in vesicular transport](../../../../I:%5CSesamum_indicum%5CSesamum_indicum%5Cannotation%5CKEGG%5CAll-Unigene.fa.htm#gene72%23gene72) | 113 | ko00604 |
| 73 | [Ether lipid metabolism](../../../../I:%5CSesamum_indicum%5CSesamum_indicum%5Cannotation%5CKEGG%5CAll-Unigene.fa.htm#gene73%23gene73) | 112 | ko00730 |
| 74 | [Ubiquinone and other terpenoid-quinone biosynthesis](../../../../I:%5CSesamum_indicum%5CSesamum_indicum%5Cannotation%5CKEGG%5CAll-Unigene.fa.htm#gene74%23gene74) | 110 | ko00905 |
| 75 | [Protein export](../../../../I:%5CSesamum_indicum%5CSesamum_indicum%5Cannotation%5CKEGG%5CAll-Unigene.fa.htm#gene75%23gene75) | 109 | ko00902 |
| 76 | [Glucosinolate biosynthesis](../../../../I:%5CSesamum_indicum%5CSesamum_indicum%5Cannotation%5CKEGG%5CAll-Unigene.fa.htm#gene76%23gene76) | 108 | ko00300 |
| 77 | [Selenoamino acid metabolism](../../../../I:%5CSesamum_indicum%5CSesamum_indicum%5Cannotation%5CKEGG%5CAll-Unigene.fa.htm#gene77%23gene77) | 107 | ko00790 |
| 78 | [Phenylalanine, tyrosine and tryptophan biosynthesis](../../../../I:%5CSesamum_indicum%5CSesamum_indicum%5Cannotation%5CKEGG%5CAll-Unigene.fa.htm#gene78%23gene78) | 103 | ko00430 |
| 79 | [N-Glycan biosynthesis](../../../../I:%5CSesamum_indicum%5CSesamum_indicum%5Cannotation%5CKEGG%5CAll-Unigene.fa.htm#gene79%23gene79) | 101 | ko00750 |
| 80 | [Diterpenoid biosynthesis](../../../../I:%5CSesamum_indicum%5CSesamum_indicum%5Cannotation%5CKEGG%5CAll-Unigene.fa.htm#gene80%23gene80) | 101 | ko00660 |
| 81 | [Glyoxylate and dicarboxylate metabolism](../../../../I:%5CSesamum_indicum%5CSesamum_indicum%5Cannotation%5CKEGG%5CAll-Unigene.fa.htm#gene81%23gene81) | 99 | ko00072 |
| 82 | [beta-Alanine metabolism](../../../../I:%5CSesamum_indicum%5CSesamum_indicum%5Cannotation%5CKEGG%5CAll-Unigene.fa.htm#gene82%23gene82) | 96 | ko00603 |
| 83 | [Benzoxazinoid biosynthesis](../../../../I:%5CSesamum_indicum%5CSesamum_indicum%5Cannotation%5CKEGG%5CAll-Unigene.fa.htm#gene83%23gene83) | 89 | ko00232 |
| 84 | [Regulation of autophagy](../../../../I:%5CSesamum_indicum%5CSesamum_indicum%5Cannotation%5CKEGG%5CAll-Unigene.fa.htm#gene84%23gene84) | 82 | ko00785 |
| 85 | [Steroid biosynthesis](../../../../I:%5CSesamum_indicum%5CSesamum_indicum%5Cannotation%5CKEGG%5CAll-Unigene.fa.htm#gene85%23gene85) | 80 | ko00780 |
| 86 | [Valine, leucine and isoleucine biosynthesis](../../../../I:%5CSesamum_indicum%5CSesamum_indicum%5Cannotation%5CKEGG%5CAll-Unigene.fa.htm#gene86%23gene86) | 79 | ko00965 |
| 87 | [Other glycan degradation](../../../../I:%5CSesamum_indicum%5CSesamum_indicum%5Cannotation%5CKEGG%5CAll-Unigene.fa.htm#gene87%23gene87) | 78 | ko00062 |
| 88 | [Sulfur metabolism](../../../../I:%5CSesamum_indicum%5CSesamum_indicum%5Cannotation%5CKEGG%5CAll-Unigene.fa.htm#gene88%23gene88) | 78 | ko00950 |
| 89 | [Histidine metabolism](../../../../I:%5CSesamum_indicum%5CSesamum_indicum%5Cannotation%5CKEGG%5CAll-Unigene.fa.htm#gene89%23gene89) | 70 | ko03450 |
| 90 | [Glycosaminoglycan degradation](../../../../I:%5CSesamum_indicum%5CSesamum_indicum%5Cannotation%5CKEGG%5CAll-Unigene.fa.htm#gene90%23gene90) | 55 | ko00960 |
| 91 | [Isoquinoline alkaloid biosynthesis](../../../../I:%5CSesamum_indicum%5CSesamum_indicum%5Cannotation%5CKEGG%5CAll-Unigene.fa.htm#gene91%23gene91) | 51 | ko00670 |
| 92 | [Non-homologous end-joining](../../../../I:%5CSesamum_indicum%5CSesamum_indicum%5Cannotation%5CKEGG%5CAll-Unigene.fa.htm#gene92%23gene92) | 51 | ko00563 |
| 93 | [Tropane, piperidine and pyridine alkaloid biosynthesis](../../../../I:%5CSesamum_indicum%5CSesamum_indicum%5Cannotation%5CKEGG%5CAll-Unigene.fa.htm#gene93%23gene93) | 45 | ko00942 |
| 94 | [One carbon pool by folate](../../../../I:%5CSesamum_indicum%5CSesamum_indicum%5Cannotation%5CKEGG%5CAll-Unigene.fa.htm#gene94%23gene94) | 45 | ko00760 |
| 95 | [Glycosylphosphatidylinositol(GPI)-anchor biosynthesis](../../../../I:%5CSesamum_indicum%5CSesamum_indicum%5Cannotation%5CKEGG%5CAll-Unigene.fa.htm#gene95%23gene95) | 43 | ko00770 |
| 96 | [Anthocyanin biosynthesis](../../../../I:%5CSesamum_indicum%5CSesamum_indicum%5Cannotation%5CKEGG%5CAll-Unigene.fa.htm#gene96%23gene96) | 43 | ko00590 |
| 97 | [Nicotinate and nicotinamide metabolism](../../../../I:%5CSesamum_indicum%5CSesamum_indicum%5Cannotation%5CKEGG%5CAll-Unigene.fa.htm#gene97%23gene97) | 40 | ko00901 |
| 98 | [Pantothenate and CoA biosynthesis](../../../../I:%5CSesamum_indicum%5CSesamum_indicum%5Cannotation%5CKEGG%5CAll-Unigene.fa.htm#gene98%23gene98) | 39 | ko00196 |
| 99 | [Arachidonic acid metabolism](../../../../I:%5CSesamum_indicum%5CSesamum_indicum%5Cannotation%5CKEGG%5CAll-Unigene.fa.htm#gene99%23gene99) | 37 | ko00523 |
| 100 | [Indole alkaloid biosynthesis](../../../../I:%5CSesamum_indicum%5CSesamum_indicum%5Cannotation%5CKEGG%5CAll-Unigene.fa.htm#gene100%23gene100) | 37 | ko00740 |
| 101 | [Photosynthesis - antenna proteins](../../../../I:%5CSesamum_indicum%5CSesamum_indicum%5Cannotation%5CKEGG%5CAll-Unigene.fa.htm#gene101%23gene101) | 35 | ko00604 |
| 102 | [Polyketide sugar unit biosynthesis](../../../../I:%5CSesamum_indicum%5CSesamum_indicum%5Cannotation%5CKEGG%5CAll-Unigene.fa.htm#gene102%23gene102) | 34 | ko00730 |
| 103 | [Riboflavin metabolism](../../../../I:%5CSesamum_indicum%5CSesamum_indicum%5Cannotation%5CKEGG%5CAll-Unigene.fa.htm#gene103%23gene103) | 34 | ko00905 |
| 104 | [Glycosphingolipid biosynthesis - ganglio series](../../../../I:%5CSesamum_indicum%5CSesamum_indicum%5Cannotation%5CKEGG%5CAll-Unigene.fa.htm#gene104%23gene104) | 33 | ko00902 |
| 105 | [Thiamine metabolism](../../../../I:%5CSesamum_indicum%5CSesamum_indicum%5Cannotation%5CKEGG%5CAll-Unigene.fa.htm#gene105%23gene105) | 33 | ko00300 |
| 106 | [Brassinosteroid biosynthesis](../../../../I:%5CSesamum_indicum%5CSesamum_indicum%5Cannotation%5CKEGG%5CAll-Unigene.fa.htm#gene106%23gene106) | 32 | ko00790 |
| 107 | [Monoterpenoid biosynthesis](../../../../I:%5CSesamum_indicum%5CSesamum_indicum%5Cannotation%5CKEGG%5CAll-Unigene.fa.htm#gene107%23gene107) | 31 | ko00430 |
| 108 | [Lysine biosynthesis](../../../../I:%5CSesamum_indicum%5CSesamum_indicum%5Cannotation%5CKEGG%5CAll-Unigene.fa.htm#gene108%23gene108) | 28 | ko00750 |
| 109 | [Folate biosynthesis](../../../../I:%5CSesamum_indicum%5CSesamum_indicum%5Cannotation%5CKEGG%5CAll-Unigene.fa.htm#gene109%23gene109) | 22 | ko00660 |
| 110 | [Taurine and hypotaurine metabolism](../../../../I:%5CSesamum_indicum%5CSesamum_indicum%5Cannotation%5CKEGG%5CAll-Unigene.fa.htm#gene110%23gene110) | 21 | ko00072 |
| 111 | [Vitamin B6 metabolism](../../../../I:%5CSesamum_indicum%5CSesamum_indicum%5Cannotation%5CKEGG%5CAll-Unigene.fa.htm#gene111%23gene111) | 19 | ko00603 |
| 112 | [C5-Branched dibasic acid metabolism](../../../../I:%5CSesamum_indicum%5CSesamum_indicum%5Cannotation%5CKEGG%5CAll-Unigene.fa.htm#gene112%23gene112) | 18 | ko00232 |
| 113 | Synthesis and degradation of ketone bodies | 17 | ko00785 |
| 114 | [Glycosphingolipid biosynthesis - globo series](../../../../I:%5CSesamum_indicum%5CSesamum_indicum%5Cannotation%5CKEGG%5CAll-Unigene.fa.htm#gene114%23gene114) | 17 | ko00780 |
| 115 | [Caffeine metabolism](../../../../I:%5CSesamum_indicum%5CSesamum_indicum%5Cannotation%5CKEGG%5CAll-Unigene.fa.htm#gene115%23gene115) | 15 | ko00965 |
| 116 | [Lipoic acid metabolism](../../../../I:%5CSesamum_indicum%5CSesamum_indicum%5Cannotation%5CKEGG%5CAll-Unigene.fa.htm#gene116%23gene116) | 12 | ko00062 |
| 117 | [Biotin metabolism](../../../../I:%5CSesamum_indicum%5CSesamum_indicum%5Cannotation%5CKEGG%5CAll-Unigene.fa.htm#gene117%23gene117) | 12 | ko00950 |
| 118 | [Betalain biosynthesis](../../../../I:%5CSesamum_indicum%5CSesamum_indicum%5Cannotation%5CKEGG%5CAll-Unigene.fa.htm#gene118%23gene118) | 6 | ko03450 |
| 119 | [Fatty acid elongation in mitochondria](../../../../I:%5CSesamum_indicum%5CSesamum_indicum%5Cannotation%5CKEGG%5CAll-Unigene.fa.htm#gene119%23gene119) | 6 | ko00960 |
