# Supplementary material for: Characterization of the sesame (Sesamum indicum L.) global transcriptome using Illumina paired-end sequencing and development of EST-SSR markers
Source: BMC Genomics. 2011 Sep 19;12:451. doi: 10.1186/1471-2164-12-451 (PMC3184296; doi:10.1186/1471-2164-12-451)
Supplement: Additional file 4 — Sesame germplasms for polymorphism validation with EST-SSRs. [file 1471-2164-12-451-S4.DOC]

**Additional file 4:**

**Sesame germplasms used for detecting the polymorphism of EST-SSR**

| No. | Accession Name | Accession No. | Source |
| --- | --- | --- | --- |
| 1 | Zhongzhi No.16 | ZZM5419 | Wuhan, Hubei, China |
| 2 | Luozhi No.18 | zzm4783 | Luohe, Henan, China |
| 3 | White sesame | ZZM2541 | Zhushan, Hubei, China |
| 4 | Yuzhi No.4 | ZZM3410 | Zhumadian, Henan, China |
| 5 | Zhongzhi No.7 | zzm1321 | Wuhan, Hubei, China |
| 6 | Jizhi No.1 | zzm0146 | Shijiazhuang, Hebei, China |
| 7 | Jincou sesame | ZZM3838 | Shangrao, Jiangxi, China |
| 8 | Aijiao sesame | ZZM2751 | Pan’an, Zhejiang, China |
| 9 | Margo | ZZM1550 | America |
| 10 | Determinate sesame No.8 | WZM4242 | America |
| 11 | U.C.R/82No15NS | WZM4220 | America |
| 12 | Mishuo sesame | ZZM2748 | Dongyang, Zhejiang,China |
| 13 | Duoleng sesame | ZZM3395 | Pucheng, Shanxi, China |
| 14 | Grey sesame | ZZM2192 | Shangnan, Shanxi, China |
| 15 | Sesame | ZZM3973 | Yueyang, Hunan, China |
| 16 | Qiuheixuan | ZZM3926 | Shangrao, Jiangxi, China |
| 17 | Soviet Russia oil crop institute No.1 | WZM1510 | Soviet Russia |
| 18 | NW1 | WZM4251 | Thailand |
| 19 | 0679 | - | Venezuela |
| 20 | Brown sesame | WZM1520 | Burma |
| 21 | Teras-<1> | WZM3069 | Mexico |
| 22 | Inamar | WZM3120 | Mozambique |
| 23 | 91-2003 | WZM4480 | Israel |
| 24 | Jinan | WZM4236 | South Korea |
